# Creating and Validating the DESEA Questionnaire for Men and Women

**DOI:** 10.3390/jcm9072301

**Published:** 2020-07-20

**Authors:** Francisco Cabello-Santamaría, Marina A. Cabello-García, Jerónimo Aragón-Vela, F. Javier del Río

**Affiliations:** 1 Instituto Andaluz de Sexología y Psicología, 29001 Málaga, Spain; fcabello@iasexologia.com (F.C.-S.); marina_cabello@hotmail.com (M.A.C.-G.); 2 Department of Nutrition, Exercise, and Sport (NEXS), University of Copenhagen, DK-1017 Copenhagen K, Denmark; jeroav@ugr.es; 3 Departamento de Psicología, Facultad de Ciencias de la Educación, Campus de Puerto Real, Universidad de Cádiz, 11003 Cádiz, Spain

**Keywords:** DESEA questionnaire, sexual desire, psychometric properties, confirmatory factor analysis

## Abstract

In clinical practice, it is essential to be able to identify hypoactive sexual desire disorder (HSDD), with its different severity levels and assess the influence the subject’s relationship has on the issue. In order to do this, questionnaires are needed that comprise appropriate psychometric properties. We analyzed the psychometric properties and factorial structure of the Sexual Desire and Aversion (DESEA) questionnaire that evaluates sexual desire and interpersonal stress (relationship problems) in male and female couples. A pilot study was conducted with a group of 1583 people. Finally, it included 20,424 Spanish speakers who answered the questionnaire via an online link. The requirements for factor analysis were verified followed by the exploratory and confirmatory factor analysis. The Cronbach’s alpha coefficient calculated the reliability of the test scores at 0.834 in the pilot group and 0.889 in the final group. A 3-factor factorial design explains the 62.08% variance. The KMO (Kaiser-Meyer-Olkin) test (*p* = 0.904), Bartlett’s test of sphericity (126,115.3; *p* = 0.000010) and the matrix determinant (0.0020770) verified the appropriateness of the factor analysis. The results show that the DESEA questionnaire is a reliable and valid instrument for evaluating desire and interpersonal stress, both in women and men, in clinical and research contexts.

## 1. Introduction

Sexual desire is commonly defined as the subjective psychological status to initiate and maintain human sexual behavior, triggered by internal and/or external stimuli [1,2]. Sexual desire has three biopsychosocial components: Drive, the biological aspect including anatomy and physiology of the neuroendocrine system; motivation, the psychological aspect, including mental state, relational issues, and social context; and wish, the cultural element considering ideals, values, and rules regarding the expression of sexuality [3].

In The Diagnostic and Statistical Manual of Mental Disorders, DSM-5 [4], female hypoactive sexual desire disorder and female arousal disorder have disappeared in order to be combined under the term “Sexual Interest/Arousal Disorder”. Sexual aversion disorder has also been removed from DSM-5 [5,6], and the hypoactive sexual desire disorder in the male is described [4].

However, distancing itself from the DSM-5, the International Society for the Study of Women’s Sexual Health defines hypoactive sexual desire disorder (HSDD) as a separate disorder to arousal disorder [7]. In the new International Society for the Study of Women’s Sexual Health (ISSWSH) nomenclature, HSDD presents with any of the following characteristics for a minimum period of at least six months: Lack of motivation for sexual activity characterized by decreased or absent spontaneous desire (i.e., sexual thoughts or fantasies); decreased or absent responsive desire to erotic cues or stimulation or inability to maintain desire or interest through sexual activity and loss of desire to initiate or participate in sexual activity, including behavioral responses such as avoiding situations that could lead to sexual activity that is not secondary to sexual pain disorders [4].

This is also the case with the CIE-11 which persists in defining it as an independent diagnosis [8].

This study does not look to evaluate if the changes in the DSM-5 were correct or not; but rather it seeks to develop a tool which can be used to differentiate between distinct treatment options in order to better address the different clinical forms that present in daily practice.

At present, all studies on the prevalence of sexual disorders recognize that HSDD is among the most common [9,10,11]. On the other hand, research on HSDD in men is scarce [12], but some studies have reported a prevalence of up to 28% [13] and is clear that male sexual desire is characterized by an interplay among biological, psychological, sexual, relational, and cultural elements [14]. Male Hypoactive Sexual Desire Disorder is defined in the DSM-5 as persistent or recurrently deficient sexual or erotic thoughts, fantasies, and desire for sexual activity. These symptoms must have persisted for a minimum of six months, and they must cause clinically significant distress [4].

There are already various validated questionnaires in Spanish to exclusively evaluate HSDD [15], such as the Inhibited Sexual Desire Test [16], and the Sexual Desire Inventory [17]. There are also questionnaires which evaluate various aspects of sexual functioning which include sexual desire. For example, the Massachusetts General Hospital Sexual Functioning Questionnaire [18], the Female Sexual Function Index [19], or Changes in Sexual Functioning Questionnaire [20]. It is important to note that these questionnaires were originally designed in English and their validation in some cases presents serious limitations [21]. Therefore, there are no validated Spanish questionnaires which evaluate the HSDD at the same time as the interaction between the couple, an aspect which is also lacking from the aforementioned instruments which evaluate sexual desire as another stage in sexual functioning. For this reason, the DESEA questionnaire has been designed. The Sexual Desire and Aversion (DESEA) questionnaire aims to overcome methodological limitations through various aspects. First, strictly following the specifications of the specialized literature on the design of psychological measurement instruments. Secondly, using a number of appropriated participants according to the aim of the study. In addition, statistical tests not carried out in previous validations have been used. Finally, linguistic and cultural translation has been designed in the source language, not from a questionnaire. It is a quick and simple questionnaire and it intends to be a useful tool in clinical practice as it can differentiate between three distinct ways of intervening when confronted with a lack of desire or when someone fulfils the criteria for HSDD (women) of ISSWSH nomenclature and/or Hypoactive Sexual Desire (men; DSM-5). It recognizes if the HSDD is due to interpersonal issues. In this case, a therapeutic approach should be taken to improve communication in addition to other therapeutic elements to improve the situation between the two individuals. If the absence of desire is due to internal desire triggers, the approach to be taken should pursue existing sexual therapy models such as, for example, the model designed by Trudel, Ravart, and Aubin [22], the models Brotto et al. [23], Cabello-Santamaría [24], Robinson, Munns, Weber-Main, Lowe, and Raymond [25] among others. Finally, if an aversive component appears accompanied by feelings of disgust and distaste to sex, interventions that are either based on Exposure Response Prevention [8] or systematic desensitization should be taken in addition to any of the previous therapeutic models. This technique has been proved effective in several studies on treating these types of clinical cases [26], although other authors do not agree with the efficacy [27].

However, serious methodological limitations [21] were showed by the previously validated Spanish questionnaires which evaluate hypoactive sexual desire [15]. Different authors pointed out as main limitations the process of cultural adaptation, insufficient sample for the purposes of validation, low power of the performed statistical tests, and that factor invariance is not usually performed [21]. There are also questionnaires which evaluate various aspects of sexual functioning which include sexual desire, but it is important to note that these questionnaires were originally designed in English and their validation in some cases presents serious limitations [21] and none of them assesses male desire. Hence, there are no validated Spanish questionnaires which evaluate the HSDD at the same time as the interaction between the couple, an aspect which is also lacking from the aforementioned instruments which evaluate sexual desire as another stage in sexual functioning. For this reason, the DESEA questionnaire has been designed. Therefore, the main purpose of this study is to design and evaluate the psychometric properties in the Sexual Desire and Aversion Questionnaire (DESEA).

## 2. Method

### 2.1. Procedure

This research uses an instrumental methodology and follows on from the work of del Río, Cabello-García and Cabello-Santamaría [28]. It also follows international recommendations set out in specialized psychometric literature [29,30]. Following a bibliographic search and the working definitions of the concepts to be evaluated, 20 items were designed. Two groups were used to validate this study. According to detect possible errors in the items, a pilot group (1583 participants) was used to check the operation of the test, analyzing the psychometric properties of the items, as well as the construct validity. In the final group (20,424 individuals), the most powerful statistical analyses were performed. The items were sent to a group of 5 experts (3 psychologists and 2 medical experts in sexology) for their evaluation. They recommended the removal of 3 items. The remaining 17 items were evaluated in a pilot group and this process led to the removal of a further 3 items because they did not have adequate psychometric characteristics. Finally, the remaining 14 items were analyzed in the final group.

The participants were able to respond to the questionnaire via links on the International Academy of Medical Sexology webpage and that of the Instituto Andaluz de Sexología y Psicología (Andalusian Institute of Sexology and Psychology). Written informed consent was obtained from all participants prior to study. Data was collected via Google Drive. The study was approved by the Committee for research involving human subjects at the “ Comité de Ética de la Investigación de Cádiz (Hospital Universitario Puerta del Mar, Cádiz, Spain), Andalusian Institute of Sexology and Psychology (Malaga, Spain) and accordance with ethical principles for medical research with human beings as established in the Declaration of Helsinki by the World Medical Association and Law 15/2007 of Biomedical Research, the Personal Data Protection Act 15/1999, Good Clinical Practical principles and requirements set by regulating authorities for verifying original documents and auditing and/or inspecting research.

### 2.2. Participants

The pilot group was made up of 1583 individuals. The final group was made up of 20,424 individuals. Sociodemographic data is shown in Table 1.

### 2.3. Instruments

The Revised Sexual Opinion Survey (Encuesta Revisada de Opinión Sexual) [31]. This survey evaluates attitudes towards sexuality on the erotophobia-erotophilia continuum. It has 20 items which are answered on a Likert scale (1–7) ranging from totally disagree to totally agree. Scoring ranges from 0 (maximum erotophobia) to 120 (maximum erotophilia). The authors report a Cronbach’s alpha of 0.851.

Changes in Sexual Functioning Questionnaire (CSFQ-14) [20]. This questionnaire evaluates sexual functioning, grouping items according to the three phases of the sexual cycle (Desire, items 1 to 5; Arousal, items 7 to 9; and Orgasm, items 11 to 13). In our case, given the research characteristics, only the items which evaluate the desire phase have been used. It is made up of 14 items which are answered on a Likert scale with 5 options for answers. In the Spanish version validation by Bobes et al. [32], they observed reliability coefficients for the aforementioned areas ranging from 0.75 to 0.82. 

DESEA Questionnaire (see Appendix A, Table A1). This questionnaire evaluates three different areas, Sexual Desire, Sexual Aversion, and Interpersonal Stress. It is made up of 14 items which are answered on a Likert scale with 5 options for each response (Not at all; Slightly; Moderately, Very; Extremely). The items 1 and 2 are scored from 0 to 4, and the other items score from 4 to 0. The final result (both for the whole scale and for each of the areas separately) is obtained by adding up the scores from each of the items. The questionnaire scoring ranges from 0 to 56. The scoring cut-offs are as follows: Serious hypoactive sexual desire disorder 0–5, moderate 6–8, slight 9–11; serious sexual HSDD (aversion) 0–4, moderate 5–6, slight 7–8; serious interpersonal stress 0–5, moderate 6–10, slight 11–15. A person is considered to have a dysfunctional score if their overall score is equal to or less than 34. The transition from direct to clinical scores was based on the criteria of the experts group.

### 2.4. Statistical Analysis

Descriptive analysis was carried out on the group and items (mean, standard deviation), as well as the corrected item-total correlation, Cronbach’s alpha (both for the questionnaire as a whole and for each of the areas), in the pilot group and in the final number of participants. Furthermore, for the pilot group, the correlation between scoring for the DESEA questionnaire scales and scoring obtained in the other two questionnaires was examined in order to evaluate the concurrent validity of the questionnaire. For the final group, the Pearson correlation was calculated between the areas and the questionnaire items. All these calculations were carried out with the IT program IBM© SPSS© Statistics Version 19 (SPSS, version 19 for Windows; IBM Corp., Armonk, NY, USA). Finally, in the final group the requisites for factor analysis were calculated (Kaiser-Meyer-Olkin test, KMO; Barlett’s test of sphericity; the matrix determinant) as well as the exploratory and confirmatory factor analysis and indices that calculate adjustments: CFI (Comparative Fix Index), RMSEA (Root Mean Square Error of Approximation), NNFI (Non-Normed Fit Index), and GFI (Goodness of Fit Index), through the program, FACTOR [33]. The graphic form of the Confirmatory Factor Analysis was created using the program, EQS version 6.1 (EQS, version 6.1 for Windows; Multivariate Software, Inc., Broadway, CA, USA) [34]. Factor invariance was calculated using a specific program (AMOS).

## 3. Results

### 3.1. Pilot Study

When the items were analyzed, three items received scoring between 0.140 and 0.195 while the others obtained scoring between 0.312 and 0.675. It was decided that items that did not meet a corrected item-total correlation of over 0.300 would be removed. The Cronbach’s alpha obtained for the items which made up the definitive version of the questionnaire was 0.834, which was considered appropriate according to the literature.

Due to the fact that the variables did not comply with the normal distribution (DESEA: Z = 4.735, *p* = 0.000; EROS: Z = 3.077, *p* = 0.000; CSFQ-14: Z = 3.240, *p* = 0.000), it was decided that the Spearman correlation should be carried out to verify the concurrent validity of the DESEA questionnaire. The CSFQ-14 questionnaire scoring correlated statistically with the DESEA questionnaire’s scale of desire (0.364, *p* = 0.000), scale of aversion (0.116, *p* = 0.000), and scale of interpersonal stress (0.013, *p* = 0.000). This means that those people who score highly on the desire scale of the CSFQ-14 questionnaire will obtain a high score on the desire scale in the DESEA questionnaire. The same is true for the aversion scale (indicative of low levels of aversion) and on the interpersonal stress scale (indicative of low levels of interpersonal stress). In turn, the EROS survey correlates with the desire scale (0.206, *p* = 0.000), the aversion scale (0.098, *p* = 0.001), and the interpersonal stress scale (0.050, *p* = 0.000). This means that people who obtain a high score of erotophilia will obtain a high score in the DESEA questionnaire in desire, a high score in aversion (indicating low levels of aversion) and highly in interpersonal stress (indicating low levels of interpersonal stress).

The KMO tests (0.865), Bartlett’s test of sphericity (7350.956; *p* = 0.000) and the matrix determinant (0.008) verified the appropriateness of the factor analysis. Exploratory factor analysis was carried out using the matrix of polychoric correlations, using the unweighted least squares extraction method and Promin oblique rotation, which is the most recommended in this case [35]. A 3-factor factorial design explains the 56.62% variance.

### 3.2. Final Group

First, the items were analyzed in order to verify that they all had values that were appropriate enough to be included in further analysis. Table 2 shows the item-total correlation and descriptive scoring for each item from the questionnaire’s definitive version. The reliability of the scale was assessed using Cronbach’s alpha coefficient, whose value was 0.889. According to Nunnally and Bernstein’s recommendations [36], the questionnaire displays high levels of internal consistency. The KMO test (0.904), Bartlett’s test of sphericity (126,115.3; *p* = 0.000010) and the matrix determinant (0.0020770) were carried out to verify the appropriateness of the factor analysis. The tests confirm the appropriateness of the factor analysis.

The exploratory factor analysis was carried out through the matrix of polychoric correlations, using the unweighted least squares extraction method and Promin oblique rotation. A 3-factor factorial design explains the 62.08% variance. The items which load on each one of the factors are shown in Table 3. The cut-off point for assigning an item to a factor was established when loading was equal to or more than 0.4.

The factors correspond accurately to the theoretical components designed: F1 Desire, F2 Sexual Aversion, F3 Interpersonal Stress. In order to evaluate the reliability of each of the factors, the Cronbach’s alpha was calculated for each, obtaining the following results: F1 = 0.754, F2 = 0.643, F3 = 0.887. As expected, the factor which obtained the lowest score is factor 2 which is also the one that has the least number of items.

Following this factor structure and the cut-off points established by the criteria of the group of experts, the participants were divided in 18 groups (Table 4).

Table 5 shows the correlation that exists between the different factors and the total scoring in the questionnaire. The factor which correlates most closely with the overall questionnaire score is Interpersonal Stress, followed by the Desire factor and then Aversion which also corresponds to the number of items belonging to each. It can be understood as a methodological artefact. Table 6 shows the correlation between the different items in the questionnaire. Correlations range from 0.171 to 0.700 with 0.01 being significant in all.

**Figure 1:**
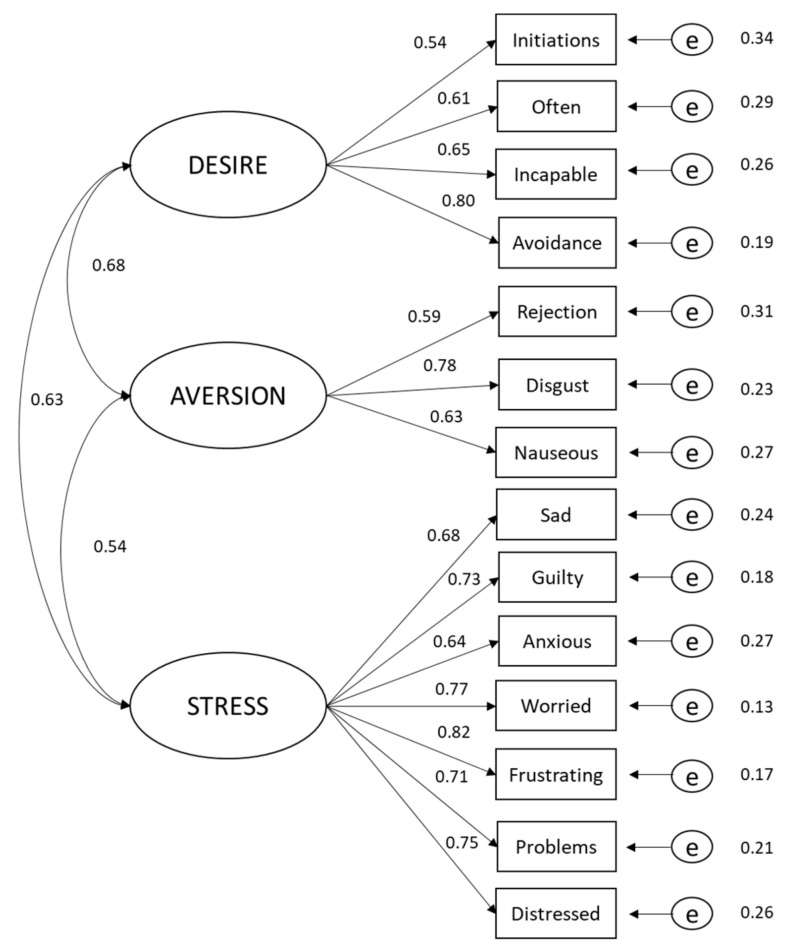
Confirmatory factor analysis model of three factors of the first order. One-headed arrows represent standardized factor loadings and two-headed arrows represent correlations. All coefficients are significant at the *p* = 0.005 level.

The confirmatory factor analysis confirmed the factor structure of the questionnaire, given that global fit indices showed adequate data in the model: CFI = 0.981, RMSEA = 0.07, NNFI = 0.967, GFI = 0.994. Figure 1 shows a graphic form of the confirmatory factor analysis. Factor invariance was calculated in the male and female groups. The RMSEA and its 90% confidence interval showed that the three-factor model predicted the data matrix in sex group (Configural: RMSEA = 0.071 [0.070–0.072], CFI = 0.983; Weak: RMSEA = 0.070 [0.069–0.072], CFI = 0.981; Strong: RMSEA = 0.070 [0.069–0.072]; CFI = 0.981). The outcomes showed that the same factor structure was conserved in both men and women.

As statistically significant differences presented (Mann–Whitney U test = 15368405.50; *p* = 0.000) in the mean scores among men (M = 45.42, SD = 7.99) and women (M = 43.08, SD = 9.81), the decision was taken to establish a scale in percentiles for men and another for women (see Table 7). The group of women ranged from a score of 13 (percentile 1) to 56 (percentile 99) and the men ranged from 20 (percentile 1) to 56 (percentile 99). The asymmetry index was negative both in men (−1.226), and women (−1.133) indicating that scoring was positioned to the right of the distribution. The Kurtosis index was greater than 0 both in men (1.549) and in women (0.902) indicating that the distribution follows a Leptokurtic curve.

## 4. Discussion

The aim of this research was to analyze the psychometric properties and the factorial structure of the new DESEA questionnaire. Due to the prevalence of HSDD, a reliable and valid assessment instrument is necessary both when working with women and men.

First, a pilot study was conducted with a group of 1583 people. Using the item-total correlation data, the items were analyzed and as a result 14 items remained in the questionnaire which obtained sufficient levels of reliability (0.834). The validity of the questionnaire was also evident in the pilot group as the scorings correlated with other previously validated questionnaires. This supports the interpretation of the DESEA questionnaire scoring in the sense that it highlights the theory that underpins it. The factor analysis carried out with the group pilot confirms the 3-factor structure of the questionnaire, explaining the 56.62% variance.

Following this, calculations were carried out on the final number of participants (20,424 individuals) to validate the questionnaire. The ability to discriminate between items was analyzed by correlating the scorings obtained by the subjects in the item with that obtained in the test overall. The correlations ranged from 0.381 to 0.681 with 85.71% being above 0.460, which confirms an adequate correlation according to Ebel standards [37]. It can therefore be observed that all the items are appropriate in terms of how they discriminate with overall scoring, which favors high levels of reliability in the questionnaire. Reliability was analyzed with Cronbach’s alpha, obtaining a score of 0.889 which means that the questionnaire was considered appropriate for both research and clinical practice [36].

Following this, the internal structure of the questionnaire was analyzed using the exploratory factor analysis and the confirmatory factor analysis. Necessary tests were performed to verify the appropriateness of the factor analysis: KMO (0.904), Bartlett’s test of sphericity (126115.3; *p* = 0.000010) and the matrix determinant (0.0020770). In each case, the appropriateness of the factor analysis was confirmed. As the format of the questionnaire used Likert style responses with 5 options for each response (0–4), an ordinal scale measurement was obtained and therefore data was factored as recommended [38] using the matrix of polychoric correlations. As it was considered that the questionnaire’s factors are correlated, the decision was taken to carry out an oblique rotation, specifically the Promin. The exploratory factor analysis determined the existence of three factors explaining the 62.08% variance which coincide with the theory underpinning the questionnaire with adequate reliability coefficients (desire, 0.754; sexual aversion, 0.643; interpersonal stress, 0.887). The stability of the questionnaire’s internal structure was tested through the confirmatory factor analysis as recommended by Carretero-Dios and Pérez [39] resulting in a good fit for the model. The analysis of the invariance indicates that both groups, men and women, fulfill the same factorial structure, which means that the score pointed out that the structure of three factors grouped into the same items is maintained in both sexes. Other researchers have found data on the prevalence of hypoactive sexual desire that range between 21.8% and 38.8% [6], in accordance with those found in this work [9].

This research and the validation of the DESEA questionnaire is important for two reasons. On the one hand, it establishes a reliable and valid tool to measure HSDD and interpersonal stress in a couple’s relationship which is extremely useful for clinical practice. In this case, a therapeutic approach should be taken to improve communication in addition to other therapeutic elements to improve the situation between the two individuals to deal with relationship issues and unmet relationship needs. If the absence of desire is due to internal desire triggers, the approach to be taken should pursue existing sexual therapy models such as Trudel’s model, Ravart, and Aubin [22], the models Brotto et al. [23], Cabello-Santamaría [24], Robinson, Munns, Weber-Main, Lowe, and Raymond [25] among others which try to improve sexual interest. Finally, if the absence of desire is severe and an aversive component appears accompanied by feelings of disgust and distaste to sex, interventions that are either based on Exposure Response Prevention [8] or systematic desensitization should be taken in addition to any of the previous therapeutic models. This technique has been proved effective in several studies on treating these types of clinical cases [26], although other authors do not agree with the efficacy [27].

On the other hand, it is also a tool which can be used to measure sexual desire that overcomes methodological limitations presented by existing questionnaires which none of them assesses the desire of men [21].

## 5. Conclusions

The DESEA questionnaire showed an adequate invariance by sex, allowing the questionnaire could be used properly in both sexes. The items reported a proper functioning in each ones of the dimensions. Likewise, the dimensions extracted from the questionnaire have adequate adjustment indices, which guarantee the correct evaluation of the subjects. This allows a more precise evaluation of the dimensions analyzed, beyond the simple affectation detected by other generic scales that evaluate sexual functioning.

However, different limitations must be considered in this study, such as the higher number of female participants respect to male. However, men were not limited. These differences in participation could be due to women showed a greater interest in answering the questionnaire. It is also important to take into account the potential biases which come from the fact that participation in the research was online. Despite these limitations, the results show that the DESEA questionnaire is valid, reliable and useful for evaluating sexual desire, HSDD due to interpersonal stress and sexual aversion. Further studies could analyze the test–retest reliability of the questionnaire and provide more information on the factorial structure. Another limitation could be the composition of the pilot sample where many people did not have a partner 55.97% (37.46% of homosexuals or bisexuals).

## Figures and Tables

**Table 1:** Socio-demographic data.

Parameters	Pilot Group			Final Group		
	M (SD)	*n*	%	M (SD)	*n*	%
**Age**	30.44 (10.16)			34.86 (9.71)		
**Sex**						
Men		705	44.54		1894	9.27
Women		878	55.46		18,530	92.53
**Sexual orientation**						
Heterosexual		990	60.54		18,897	92.53
Homosexual		396	25.02		450	2.20
Bisexual		197	12.44		631	3.09
Asexual		-	-		446	2.18
**Studies**						
University Studies		1008	63.68		10,151	49.70
Professional training		321	20.28		5187	25.40
Secondary education		191	12.07		4653	22.78
Primary education		63	3.97		386	1.89
Did not have any studies		-	-		47	0.23
**Political orientation**						
Left-wing		843	53.25		1479	7.25
Centre		479	30.26		2053	10.05
Right-wing		172	10.87		3324	16.27
Not to answer		89	5.62		13,568	66.43
**Relationship**						
Did not have a partner		886	55.97		1688	8.26
Occasional dates		-	-		2181	10.68
Had a partner but did not live together		-	-		4375	21.42
Married or lived with their partner		600	37.90		11,521	56.41
Divorced		53	3.35		51	0.25
Separated		36	2.27		-	-
Widowed		8	0.51		14	0.07
More than one partner		-	-		12	0.06
Without exclusive relationship		-	-		4057	19.86

M, Mean; SD, standard deviation.

**Table 2:** Mean, standard deviation, corrected item-total correlation and Cronbach’s alpha if the (*n* = 20,424) element is removed.

Items	Mean	Variance	r_i-T_	α
Initiations	1.592	1.395	0.386	0.890
Often	2.203	1.443	0.481	0.886
Incapable	3.273	0.930	0.535	0.883
Avoidance	3.075	1.237	0.628	0.879
Sad	3.234	1.356	0.663	0.877
Guilty	3.282	1.212	0.647	0.878
Anxious	3.328	1.170	0.593	0.880
Worried	2.916	1.747	0.640	0.878
Frustrating	3.124	1.371	0.681	0.876
Problems	3.259	1.232	0.642	0.878
Distressed	3.182	1.236	0.665	0.877
Rejection	3.305	1.138	0.544	0.882
Disgust	3.719	0.549	0.466	0.886
Nauseous	3.811	0.346	0.381	0.889

**Table 3:** Rotated component matrix.

Items	F1	F2	F3
Initiations	0.689		
Often	0.784		
Incapable	0.400		
Avoidance	0.475		
Sad			0.499
Guilty			0.694
Anxious			0.538
Worried			0.828
Frustrating			0.894
Problems			0.628
Distressed			0.713
Rejection		0.410	
Disgust		0.875	
Nauseous		0.630	

**Table 4:** The Sexual Desire and Aversion Questionnaire (DESEA) score for both men and women.

	Men	Women
Several hypoactive sexual desire	47 (2.48%)	1903 (10.27%)
Moderate hypoactive sexual desire	235 (12.41%)	4156 (22.43%)
Mild hypoactive sexual desire	510 (26.93%)	5809 (31.35%)
Several interpersonal stress	23 (1.21%)	470 (2.54%)
Moderate interpersonal stress	54 (2.85%)	817 (4.41%)
Mild interpersonal stress	161 (8.50%)	1452 (7.84%)
Several sexual aversion	10 (0.53%)	417 (2.25%)
Moderate sexual aversion	18 (0.95%)	379 (2.05%)
Mild sexual aversion	100 (5.28%)	1169 (6.31%)

**Table 5:** Correlations in factors and total scoring in the questionnaire (*n* = 20,424).

	Desire Factor	Aversion Factor	Interpersonal Stress Factor
Aversion Factor			
Pearson Correlation	0.478 *		
Significance (bilateral)	0.000		
Interpersonal Stress Factor			
Pearson Correlation	0.509 *	0.511 *	
Significance (bilateral)	0.000	0.000	
Total questionnaire			
Pearson Correlation	0.772 *	0.692 *	0.923 *
Significance (bilateral)	0.000	0.000	0.000

Note: * *p* < 0.01.

**Table 6:** Correlation between questionnaire items.

Items	Initiations	Often	Incapable	Avoidance	Sad	Guilty	Anxious	Worried	Frustrating	Problems	Distressed	Rejection	Disgust
Often	0.569 *												
Incapable	0.276 *	0.363 *											
Avoidance	0.401 *	0.450 *	0.546 *										
Sad	0.277 *	0.382 *	0.439 *	0.458 *									
Guilty	0.210 *	0.291 *	0.357 *	0.383 *	0.539 *								
Anxious	0.211 *	0.286 *	0.353 *	0.369 *	0.600 *	0.498 *							
Worried	0.173 *	0.245 *	0.305 *	0.331 *	0.505 *	0.565 *	0.494 *						
Frustrating	0.178 *	0.260 *	0.333 *	0.363 *	0.517 *	0.629 *	0.493 *	0.700 *					
Problems	0.196 *	0.248 *	0.310 *	0.390 *	0.399 *	0.466 *	0.366 *	0.536 *	0.571 *				
Distressed	0.189 *	0.248 *	0.334 *	0.398 *	0.445 *	0.515 *	0.436 *	0.551 *	0.618 *	0.684 *			
Rejection	0.195 *	0.238 *	0.326 *	0.424 *	0.333 *	0.351 *	0.297 *	0.385 *	0.391 *	0.507 *	0.465 *		
Disgust	0.252 *	0.270 *	0.315 *	0.457 *	0.282 *	0.235 *	0.242 *	0.223 *	0.229 *	0.350 *	0.297 *	0.423 *	
Nauseous	0.171 *	0.198 *	0.237 *	0.329 *	0.247 *	0.212 *	0.219 *	0.195 *	0.202 *	0.277 *	0.253 *	0.294 *	0.558 *

Note: * *p* < 0.01.

**Table 7:** DESEA questionnaire scales in percentiles for men and women.

Percentiles	Women	Men
1	13.00	20.00
2	17.00	22.00
3	20.00	26.00
4	22.00	28.00
5	23.00	30.00
10	28.00	34.00
15	33.00	37.00
20	35.00	40.00
25	38.00	41.75
30	40.00	43.00
35	42.00	44.00
40	43.00	45.00
45	45.00	47.00
50	46.00	47.00
55	47.00	48.00
60	48.00	49.00
65	49.00	50.00
70	50.00	50.50
75	50.00	51.00
80	51.00	52.00
85	52.00	53.00
90	53.00	54.00
95	54.00	55.00
96	54.76	55.00
97	55.00	56.00
98	55.00	56.00
99	56.00	56.00

## Appendix A

**Table A1:** DESEA Questionnaire.

Answer all the Questions with the Last Month in Mind	N	S	M	V	E
1. In the last six months, how often have you initiated any type of sexual activity with your partner?	0	1	2	3	4
2. In the last six months, how often have you felt an urge to have sex?	0	1	2	3	4
3. In the last six months, have you felt incapable of desiring sexual activity?	4	3	2	1	0
4. In the last six months, to what extent have you felt that you have wanted to avoid sexual activity?	4	3	2	1	0
5. In the last six months, have you felt sad or annoyed that you have not felt sexual desire?	4	3	2	1	0
6. In the last six months, have you felt guilty for not satisfying your partner?	4	3	2	1	0
7. In the last six months, have you felt anxious for not wanting to have sex?	4	3	2	1	0
8. In the last six months, to what extent have you felt worried about the future of your relationship with your partner because of your desire?	4	3	2	1	0
9. In the last six months, have you felt bad about the idea of frustrating your partner due to your level of desire?	4	3	2	1	0
10. In the last six months, have you had problems in your relationship due to your sexual desire?	4	3	2	1	0
11. In the last six months, to what extent have you felt distressed or anxious with your partner due to your level of sexual desire?	4	3	2	1	0
12. In the last six months, have you felt rejection towards your partner when he/she has initiated sexual contact?	4	3	2	1	0
13. In the last six months, have you felt disgust at the idea of having sex?	4	3	2	1	0
14. In the last six months, have you felt nauseous or had digestive problems or headaches at the thought of having sex or during any sexual act?	4	3	2	1	0

Instructions: Please mark your answer with a cross in the box for each of the following questions. You can respond to each of the questions in the following ways: N: Not At All; S: Slightly; M: Moderately; V: Very; E: Extremely.
